# Infective endocarditis caused by *Capnocytophaga canimorsus*; a case report

**DOI:** 10.1186/s12879-019-4492-3

**Published:** 2019-11-04

**Authors:** Jun Sakai, Kazuhito Imanaka, Masahiro Kodana, Kana Ohgane, Susumu Sekine, Kei Yamamoto, Yusuke Nishida, Toru Kawamura, Takahiro Matsuoka, Shigefumi Maesaki, Hideaki Oka, Hideaki Ohno

**Affiliations:** 1 Department of Infectious Disease and Infection Control, Saitama Medical Center, Saitama Medical University, 1981 Kamoda, Kawagoe, Saitama, 350-8550 Japan; 2 Department of General Internal Medicine, Saitama Medical Center, Saitama Medical University, Saitama, Japan; 3 Department of Infectious Disease and Infection Control, Saitama Medical University Hospital, Saitama Medical University, Saitama, Japan; 4 Department of Cardiovascular Surgery, Saitama Medical Center, Saitama Medical University, Saitama, Japan; 5 Department of Laboratory Medicine, Saitama Medical University Hospital, Saitama Medical Hospital, Saitama, Japan; 6 Department of Clinical Laboratory, Saitama Medical Center, Saitama Medical University, Saitama, Japan

**Keywords:** *Capnocytophaga canimorsus*, Ceftriaxone, Drug susceptibility test, Infective endocarditis

## Abstract

**Background:**

*Capnocytophaga canimorsus* is a gram-negative bacterium and an oral commensal in dogs and cats, but occasionally causes serious infections in humans. Septicemia is one of the most fulminant forms, but diagnosis of *C. canimorsus* infection is often difficult mainly because of its very slow growth. *C. canimorsus* infective endocarditis (IE) is rare and is poorly understood. Since quite a few strains produce β-lactamase, antimicrobial susceptibility is pivotal information for adequate treatment. We herein report a case with *C. canimorsus* IE and the results of drug susceptibility test.

**Case presentation:**

A 46-year-old man had a dog bite in his left hand 3 months previously. The patient was referred to our hospital for fever (body temperature > 38 °C), visual disturbance, and dyspnea. Echocardiography showed aortic valve regurgitation and vegetation on the leaflets. IE was diagnosed, and we initially administered cefazolin and gentamycin assuming frequently encountered microorganisms and the patient underwent aortic valve replacement. *C. canimorsus* was detected in the aortic valve lesion and blood cultures. It was also identified by 16S ribosome DNA sequencing. Ceftriaxone were started and continued because disk diffusion test revealed the isolate was negative for β-lactamase and this case had cerebral symptoms. The patient successfully completed antibiotic treatment following surgery.

**Conclusions:**

We diagnosed *C. canimorsus* sepsis and IE by extended-period blood cultures and 16S ribosome DNA sequencing by polymerase chain reaction, and successfully identified its drug susceptibility.

## Background

*Capnocytophaga canimorsus* is a gram-negative bacillus found in saliva of healthy dogs and cats and is transmitted to humans principally through animal bites [1]. It can cause sepsis and other forms of infection. Here, we report a patient with sepsis and infective endocarditis (IE) caused by *C. canimorsus*. As *C. canimorsus* IE is rare and this microbe is difficult to culture, drug susceptibility is often unclear and its standard treatment regimen remains unestablished.

## Case presentation

**Fig. 1:**
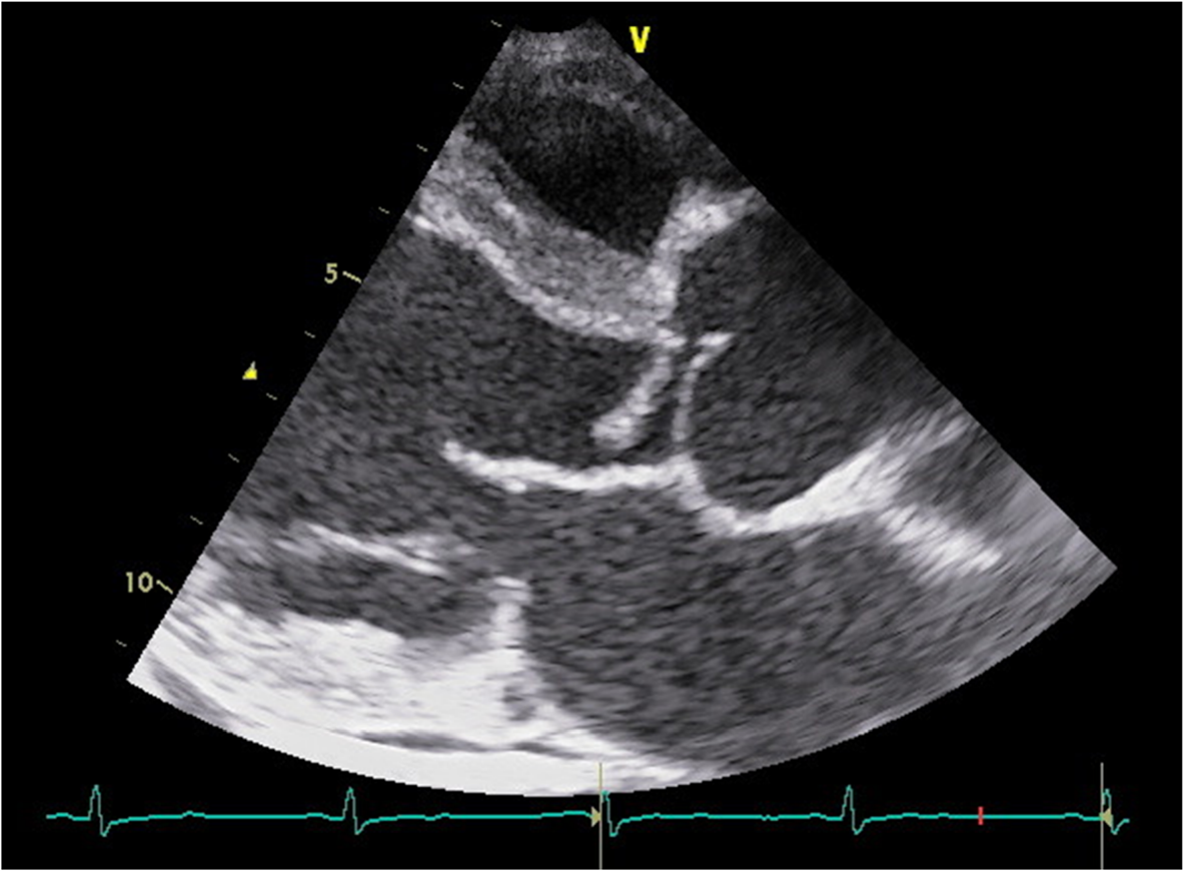
Echocardiogram showing moderate-to-severe aortic valve regurgitation and vegetation of 17-mm in size

**Fig. 2:**
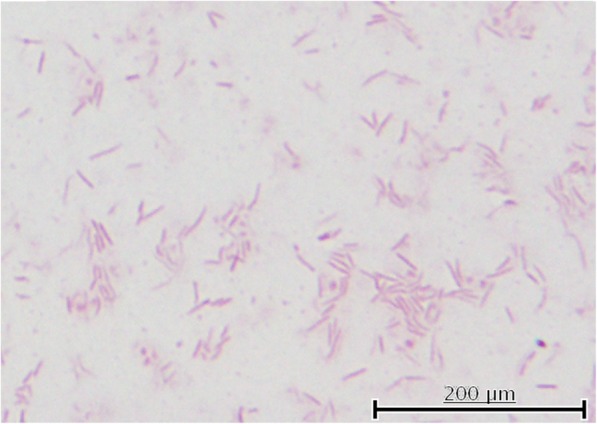
*Capnocytophaga-*like gram-negative bacilli on the aortic valve (× 1000)

A 46-year-old man with a history of dog-bite in his left hand 3 months ago, developed fever (body temperature > 38 °C), visual disturbance, and dyspnea at rest. He had been otherwise healthy without significant medical history. He was tachycardic, and coarse crackle and diastolic heart murmur (Levine III) was audible. Laboratory test results were as follows: white blood cell count, 10,500/μL (59.8% neutrophils); hemoglobin level, 11.7 g/dL; brain natriuretic protein level, 689.2 pg/mL; and C-reactive protein level, 9.0 mg/dL (Table 1). Chest X ray showed pulmonary congestion and bilateral pleural effusion. Brain magnetic resonance imaging revealed no lesion in optic nerve and brain. Transthoracic echocardiography revealed moderate-to-severe aortic valve regurgitation and vegetation of 17-mm in size (Fig. 1). Seven days later, blood culture yielded *Coagulase-negative staphylococci* in one of four culture bottles. Although diagnosis of IE was not definitive according to Duke criteria [2], history of dog bites, his clinical course, and imaging studies suggested *Staphylococcal* IE. Following administration of cefazolin 6 g/day and gentamycin 3 mg/kg/day for a week, the patient underwent aortic valve replacement and resected aortic valve was negative for Staphylococci. A week following surgery, however, microorganism grew in two bottles of preoperative blood culture. This microorganism was cultured on blood agar, and gram staining of the colonies showed *Capnocytophaga-*like gram-negative bacilli (Fig. 2). 16S ribosome DNA sequencing both from blood and from resected heart valve identified *C. canimorsus*. Disk diffusion test revealed that the isolate was susceptible to almost all antimicrobial agents and did not produce β-lactamase (Table 2). The protocol of the disk diffusion test was as follows: A Brucella HK agar plate was seeded with a lawn of *C. canimorsus* using sterile cotton swabs. For the plate, antibiotic disks containing 10 IU of penicillin G, 10 μg/10 μg of sulbactam/ampicillin, 10 μg/100 μg of tazobactam/piperacillin, 30 μg of ceftriaxone, 10 μg of meropenem, 10 μg of gentamycin, 30 μg of amikacin, 5 μg of levofloxacin, 30 μg of minocycline, 250 μg of sulfamethoxazole/trimethoprim, 15 μg of clarithromycin, 2 μg of clindamycin were used with BD Sensi-Disc (BD Bioscience Co., USA) and dispensed on the agar surface. Both plates were incubated at 30 °C overnight and the diameter of each zone was measured in millimeters to evaluate susceptibility or resistance using the comparative standard method.

**Table 1:** Laboratory data on admission

(A) Peripheal blood data	
Peripheral Blood	
WBC	10,500 /μL
Neut	59.8%
Lymp	31.1%
Mono	4.9%
Eosi	3.9%
Baso	0.3%
RBC	430 × 10^6^/μL
HCT	42.0%
Hb	11.7 g/dL
MCV	97.7 fL
MCH	32.3 pg
MCHC	33.1 pg
PLT	22.4 × 10^6^/μL
(B) Chemistry data	
Chemistry	
TP	6.3 g/dL
ALB	4.0 g/dL
AST	126 IU/L
ALT	101 IU/L
LDH	179 IU/L
γGTP	24 U/L
BUN	15 mg/dL
Cr	0.71 mg/dL
Na	142 mEq/L
K	3.3 mEq/L
Cl	107 mEq/L
CRP	9.0 mg/dL
BNP	689.2 pg/mL

*WBC* white blood cells, *Neut* neutrophils, *Lymp* lymphocytes, *Mono* monocytes, *Eosi* eosinophils, *Baso* basophils, *RBC* red blood cells, *HCT* hematocrit, *Hb* hemoglobin, *MCV* mean cell volume, *MCH* mean corpuscular hemoglobin, *MCHC* mean corpuscular hemoglobin concentration, *PLT* platelet counts, *TP* total protein, *ALB* albumin, *AST* aspartate aminotransferase, *ALT* alanine aminotransferase, *LDH* lactate dehydrogenase (upper limited: 211 IU/L), *γ-GTP* γ-glutamyl transpeptidase, *BUN* blood urea nitrogen, *Cr* creatinine, *Na* sodium, *K* potassium, *Cl* chlorine, *CRP* C-reaction peptide, *BNP* brain natriuretic protein

**Table 2:** Drug susceptibility shown by disk diffusion method

Antimicrobial Agents	Inhibition Zone (mm)
Penicillin G	32
Sulbactam/Ampicillin	36
Tazobactam/Piperacillin	38
Ceftriaxone	20
Meropenem	36
Gentamycin	< 6
Amikacin	< 6
Levofloxacin	34
Minocycline	40
Sulfamethoxazole/Trimethoprim	< 6
Clarithromycin	38
Clindamycin	34

Based on these results and symptoms, empirically selected combination of gentamycin and cefazolin was converted to ceftriaxone 4 g/day. The patient completed a total of 4 weeks of ceftriaxone. The patient has been doing well for 12 months after hospital discharge.

## Discussion and conclusion

*C. canimorsus* is a less virulent pathogen. IE accounts for less than 2% of *C. canimorsus* bloodstream infection and is extremely rare [3]. Only 18 cases have been reported in the literatures since 1977 (Table 3) [4–10]. Patients were 52.8 years of age (range 24 to 73 years) on average and were predominantly male (80.0%). Affected valves were aortic in 11 (61.1%), tricuspid in six (33.3%), and mitral in four (22.2%). Nine patients (50.0%) were surgically treated, mostly using mechanical valves. Penicillin was given in eight (44.4%), and Cephalosporin in four (22.2%). Four patients (22.2%) had underlying cardiac diseases, and five (27.7%) were vulnerable to infection; alcohol abuse in four and chronic lymphocytic leukemia undergoing chemotherapy in one. Twelve of 18 patients (66.6%) had dog bite or close contact with dogs.

**Table 3:** Infective endocarditis caused by *Capnocytophaga canimorsus* in literature

No	Age/Sex	Animal contact	Underlying disease	Infected valve	Surgery (Methods)	Antibiotics	Complications	Outcome	References
1	ND	Dog	ND	A	Yes (ND)	ND	ND	D	[4]
2	ND	ND	ND	A	No	ND	ND	S	
3	ND	ND	ND	M	No	ND	ND	S	
4	64/M	Dog	ND	T, A	No	Penicillin + Erythromycin	ND	D	
5	59/F	ND	CLL, Atrial myxoma, Steroid use	T	Yes (ND)	Cephalothin + Gentamicin	ND	D	
6	39/M	Dog	Alcohol abuse	M	No	Ampicillin + Tobramycin	Glomerulonephritis.	S	
7	24/M	Dog	None	A	No	Penicillin	ND	S	
8	47/M	Dog	Alcohol abuse	T	Yes (ND)	Vancomycin + Gentamicin	ND	S	
9	56/M	Dog	None	T	No	Penicillin + Gentamicin	ND	S	
10	52/M	Dog	None	A	No	Penicillin + Aztreonam	ND	S	
11	69/F	None	COPD	T	No	Penicillin	CHF	S	
12	63/M	Dog	AVR (Mechanical valve)	A (Periannular abscess)	Yes (AVR, Tissue valve)	Penicillin	Anemia, CHF	S	
13	41/F	Dog	Rheumatic mitral valve disease	M	Yes (MVR, Mechanical valve)	Ceftriaxone	ND	S	[5]
14	42/M	Dog	Alcohol abuse	A	Yes (AVR, Mechanical valve)	Ceftriaxon + Gentamicin	ND	S	[6]
15	55/M	Dog	COPD, Alcohol abuse, Intravenous drug user	A (Periannular abscess), T	Yes (AVR, Mechanical valve), (Aortoplasty) (Tricuspid valve repair)	Meropenem+ Ciprofloxacin	ND	S	[7]
16	65/M	None	Dislipidemia	A (Periannular abscess)	Yes (Aortic root replacement, Mechanical valve)	Ampicillin + Gentamicin	Anemia, Renal insufficiency	S	[8]
17	73/M	Dog	AVR (Mechanical valve), Diabetes, Renal insufficiency	A	No	Meropenem + Ciprofloxacin	Anemia	S	[9]
18	43/M	Lion	None	A, M	Yes (AVR, Mechanical valve) (Mitral valve annuloplasty), (Coronary artery bypass grafting)	Ceftriaxone + Gentamicin + Vancomycin	None	S	[10]

*ND* No Data, *M* Male, *F* Female, *CLL* Chronic Lymphocytic Lymphoma, *COPD* Chronic Obstructive Pulmonary Disease, *AVR* Aortic valve replacement, *A* Aortic valve, *M* Mitral valve, *T* Tricuspid valve, *MVR* Mitral valve replacement, *CHF* Congestive heart failure, *D* Died, *S* Survived

*C. canimorsus* is a facultative anaerobe and grows slowly in blood culture bottles and on agar plates. It has fastidious requirements for growth (5-10% CO_2_) and efficient culture method has not yet been established. Diagnosis of *C. canimorsus* IE generally requires high indices of suspicion because clinical symptoms are non-specific and routine blood cultures are often negative. If a pet owner or an immunocompromised host develops IE and blood culture is initially negative, therefore, longer incubation or terminal subculture should be considered. In addition, Polymerase chain reaction and sequencing for 16S rDNA is useful to identify *C. canimorsus* [11, 12].

Since IE is a life threatening illness, antibiotic treatment often needs to be commenced before causative organism is identified. Aminoglycosides and/or β-lactam antibiotics are common empirical drugs of choice. However, almost all strains of *C. canimorsus* are resistant to aminoglycosides [13]. Decades ago, β-lactamase-producing *Capnocytophaga* was less than 2% [14], but recent papers suggest such strains have remarkably increased and account for 32% [15] or 79% [16]. So far, prognosis of *C. canimorsus* IE is poor chiefly due to delay in diagnosis and suboptimal drug choice. During treatment for *C. canimorsus* IE, therefore, addition of β-lactamase-inhibitor might be beneficial. In the present case, we chose Ceftriaxone soon after extended culture yielded gram negative bacilli. As disk diffusion test showed the strain was sensitive to β-lactam antibiotics, Ceftriaxone was continued until completion.

In conclusion, *C. canimorsus* is a fastidious and slow-growing microbe. *C. canimorsus* IE shows no specific findings but this pathogen should be kept in mind especially when pet owners show fever of unknown origin. Longer incubation along with some molecular biological diagnostic methods should be considered. Because diagnosis of *C. canimorsus* IE is often delayed and β-lactam tolerance is relatively common, its prognosis is not good. Prompt antimicrobial susceptibility test is essential.

## Data Availability

All data generated or analyzed during this study are included in this published article.

## Abbreviations

IE: Infective endocarditis
